# Cleaning Windows and Paint

**Published:** 1891-12

**Authors:** 


### CLEANING WINDOWS AND PAINT.

To make more easy the cleaning of windows and paint, get a large sponge, such as is used to wash carriages, and chamois skin and go to work. Use lukewarm water. Wash off the windows, glass and frames thoroughly with a sponge, then with the skin wipe them off, and no rubbing will be required. Proceed the same with the painted work about the house, and you who try it will find your paint and windows never looked so well before. Wring the chamois as dry as you can each time you use it. One advantage of this method over the old way of cleaning is that no lint is left on paint or windows. A handy thing to have for the window corners is a tooth brush to take dust or dirt out. If the paint has been white and turned yellow, take a little soda on the sponge and rub over it, washing off with clean water, and you will be surprised to see how much better it will look. Or, if the window sill has any grease spots upon it, spread the soda thickly over them, then scrub with soap and water. One or two tablespoonfuls of ammonia added to a pail of water will clean windows better than soap.

